# Spatial autocorrelation equation based on Moran’s index

**DOI:** 10.1038/s41598-023-45947-x

**Published:** 2023-11-07

**Authors:** Yanguang Chen

**Affiliations:** Department of Geography, College of Urban and Environmental Sciences, Peking University, Beijing, 100871 People’s Republic of China

**Keywords:** Mathematics and computing, Statistics

## Abstract

Moran’s index is an important spatial statistical measure used to determine the presence or absence of spatial autocorrelation, thereby determining the selection orientation of spatial statistical methods. However, Moran’s index is chiefly a statistical measurement rather than a mathematical model. This paper is devoted to establishing spatial autocorrelation models by means of linear regression analysis. Using standardized vector as independent variable, and spatial weighted vector as dependent variable, we can obtain a set of normalized linear autocorrelation equations based on quadratic form and vector inner product. The inherent structure of the models’ parameters are revealed by mathematical derivation. The slope of the equation gives Moran’s index, while the intercept indicates the average value of standardized spatial weight variable. The square of the intercept is negatively correlated with the square of Moran’s index, but omitting the intercept does not affect the estimation of the slope value. The datasets of a real urban system are taken as an example to verify the reasoning results. A conclusion can be reached that the inner product equation of spatial autocorrelation based on Moran’s index is effective. The models extend the function of spatial analysis, and help to understand the boundary values of Moran’s index.

## Introduction

Spatial autocorrelation measures can be expressed as simple forms based on standardized vector and globally normalized weight matrix. Then the basic measures such as Moran’s index can be integrated into a pair of Eigen equations: one is inner product equation, and other is outer product equation. This two equations can be used to estimate Moran’s index and normalize Moran’s scatterplot. The scatterplot was proposed by Anselin^1^ for local spatial autocorrelation analysis. It is easy to understand and make use of the outer product equation, in which the nonzero Eigenvalue is global Moran’s index. However, the inner product equation is difficult to understand in a simple way. Formally, the Eigenvalue in the inner product equation is equal to Moran’s index. Empirically speaking, this characteristic parameter is not a numerical value, but a set of Eigenvalues. The boundary values of Moran’s index is determined by the maximum Eigenvalue and the minimum Eigenvalue. On the other hand, by means of linear regressive analysis, the characteristic parameter becomes the regressive coefficient and gives the value of Moran’s index^2^.

In fact, the inner product equation of Moran’s index deserves further research. Based on the inner product equation, two spatial autocorrelation models can be constructed. One is the linear model with a constant term, and the other is the linear model without a constant term. Thus, linear regression analysis can be employed to investigate the inner product equation of Moran’s index. Derivation of the formulae of models’ parameters is helpful for developing theory and method of spatial autocorrelation analysis. First, the results can be used to help us understand Moran’s index deeply and improve normalized Moran’s scatterplot. Second, the results can be used to analyze the boundary values of Moran’s index from different angles of view. Third, the results are useful for understanding the related spatial autoregressive modeling from the perspective of spatial autocorrelation. This paper is devoted to clarifying the mathematical structure of parameters of spatial autocorrelation models based on Moran’s index. The rest parts are organized as follows. In Sect. “Theoretical results”, two sets of parameter expressions are derived from the spatial autocorrelation models by using four methods. In Sect. “Empirical analysis”, an empirical analysis is made to verify the results of theoretical derivation. In Sect.  “Discussion”, several questions are discussed, and finally, in Sect. “Conclusion”, the discussion is concluded by summarizing the main points of this study.

## Theoretical results

### Preparatory equations

Starting from the concise mathematical form, we can derive new clear relations for spatial autocorrelation analysis. Suppose that there are *n* elements such as cities in a geographical region. We can use Moran’s index to reflect the extent of spatial autocorrelation of the *n* geographical elements^3–5^. Based on population standardized size variable and globally normalized spatial weight matrix, Moran’s index can be express as^2^

1 $$I = {\mathbf{z}}^{{\text{T}}} {\mathbf{Wz}},$$

where *I* refers to Moran’s index (Moran’s *I* for short), **z** = [*z*_1_, *z*_2_,…,* z*_*n*_]^T^ is the standardized size vector by *z*-score, **W = [***w*_*ij*_**]** is a *n* × *n* globally normalized symmetric spatial weight matrix, and the superscript T implies transposition of matrix or vector (*i*, *j* = 1,2,…,*n*). Equation (1) is a kind of quadratic form in mathematics. The main properties of globally normalized spatial weight matrix are as follows: (1) global normalization, that is, the sum of element values in **W** is 1; (2) symmetry, that is, **W**^T^ = **W**; (3) Non-negativity, that is, all the values in the matrix are greater than or equal to 0. The properties of standardized variable are as below: the mean of **z** is 0, and standard deviation of **z** is 1. Moreover, we have the following relation:

2 $${\mathbf{z}}^{{\text{T}}} {\mathbf{z}} = {\mathbf{o}}^{{\text{T}}} {\mathbf{o}} = n,$$

where **o** = [1, 1,…,1]^T^ is a one vector consisting of *n* ones. It is easy to prove Eq. (2) by using the knowledge of linear algebra or verify it by observational data.

Base on Moran’s index, a set of spatial autocorrelation equations can be constructed. The first equation is based on the outer product of **z**. Multiplying Eq. (1) left by **z** yields

3 $${\mathbf{zz}}^{{\text{T}}} {\mathbf{Wz}} = I{\mathbf{z}},$$

which is an Eigen equation of Moran’s index (Appendix 1). This suggests that the absolute value of *I* is the only nonzero Eigenvalue of the spatial correlation matrix **zz**^T^**W**, and **z** is the corresponding Eigenvector. Conversely, starting from Eq. (3), the formula of Moran index, Eq. (1), can be derived. The second equation is based on the inner product of **z**. An approximate Eigen equation can be expressed as follows

4 $${\mathbf{z}}^{{\text{T}}} {\mathbf{zWz}} = n{\mathbf{Wz}} = I{\mathbf{z}},$$

which is a pure theoretical relation. In theory, *I* is the Eigenvalue of rescaled spatial weight matrix **z**^T^**zW**, but in empirical analysis, the Eigenvalue is a set of values and can be expressed as *λ*_*i*_ (*i* = 1, 2,…, *n*). It can be proved that the value of Moran’s *I* comes between the minimum Eigenvalue *λ*_min_ and the maximum Eigenvalue *λ*_max_ of **z**^T^**zW**, that is, *λ*_min_ ≤ *I* ≤ *λ*_max_. The formula of Moran’s index, Eq. (1), can be derived from Eq. (4), but Eq. (4) cannot be derived from Eq. (1). By using the least squares calculation, we can find the following linear equation:

5 $${\mathbf{z}}^{{\text{T}}} {\mathbf{zWz}} = n{\mathbf{Wz}} = ({\mathbf{o}}^{{\text{T}}} {\mathbf{Wz}}){\mathbf{o}} + I{\mathbf{z}},$$

where **o**^T^**Wz** = (**Wz**)^T^**o** is a constant. Equations (4) and (5) are in fact inverse functions of simple spatial autoregressive models and can be regarded as inverse spatial autoregressive functions.

The above mathematical equations are helpful to improve spatial autocorrelation analysis and prepare for understanding spatial autoregressive modeling. Equations (3) and (4) can be used to generate a normalized Moran’s scatterplot^2^. Equation (4) gives scattered points, Eq. (3) gives a theoretical trend line, and both Eqs. (4) and (5) give an empirical trend lines. The slopes of the trend lines gives the Moran’s index value. However, the theoretical foundation of Eqs. (4) and (5) is not yet clear. The outer product equation of Moran’s index is easy to understand, while the corresponding inner product equation is relatively complex. For observational data from the real world, Eq. (5) should be replaced by an empirical relation as below:

6 $$n{\mathbf{Wz}} = a{\mathbf{o}} + b{\mathbf{z}} + {\mathbf{e}},$$

where *a* refers to intercept, i.e. the constant term of the regression equation, and *b* denotes the slope, i.e. the regressive coefficient in a narrow sense, **e** is a residuals set, representing an error term. A simple spatial autoregressive model can be obtained by exchanging the positions of independent variables and dependent variables in Eq. (6). Now, it is necessary to prove the follow relations: *a* = **o**^T^**Wz** = (**Wz**)^T^**o**, *b* = *I*, which are related to the theoretical essence of the Moran’s autocorrelation equations.

### Regressive coefficients of autocorrelation models

Using the principle of linear algebra and related knowledge of linear regressive analysis, we can derive the parameter expressions for the inner product equations based on Moran’s index. Equation (6) left multiplied by the transpose of vector **z** and **Wz**, respectively, yields a pair of equations as below:

7 $$n{\mathbf{z}}^{{\text{T}}} {\mathbf{Wz}} = a{\mathbf{z}}^{{\text{T}}} {\mathbf{o}} + b{\mathbf{z}}^{{\text{T}}} {\mathbf{z}} + {\mathbf{z}}^{{\text{T}}} {\mathbf{e}},$$

8 $$n({\mathbf{Wz}})^{{\text{T}}} {\mathbf{Wz}} = a({\mathbf{Wz}})^{{\text{T}}} {\mathbf{o}} + b({\mathbf{Wz}})^{{\text{T}}} {\mathbf{z}} + ({\mathbf{Wz}})^{{\text{T}}} {\mathbf{e}}.$$

The sum of the values of a standardized vector equals 0, that is, **z**^T^**o** = 0. On the other hand, the independent variables *z* and residuals *e* are orthogonal to each other, that is,

9 $${\mathbf{z}}^{{\text{T}}} {\mathbf{e}} = {\mathbf{z}}^{{\text{T}}} {\mathbf{o}} = 0.$$

The inner product between *Wz* and residuals *e* can be expressed as,

10 $$\gamma = ({\mathbf{Wz}})^{{\text{T}}} {\mathbf{e}},$$

where *γ* is a parameter and will be further explained later. According to Eqs. (1), (2) and (10), Eqs. (7) and (8) can be changed to,

11 $$nI = 0a + nb,$$

12 $$n({\mathbf{Wz}})^{{\text{T}}} {\mathbf{Wz}} - \gamma = ({\mathbf{Wz}})^{{\text{T}}} {\mathbf{o}}a + Ib.$$

From Eq. (11), the slope can be derived as,

13 $$b = I.$$

This proves that the slope is equal to Moran’s index. From Eq. (12) we derive the preliminary expression of the intercept as follows:

14 $$a = \frac{{n({\mathbf{Wz}})^{{\text{T}}} {\mathbf{Wz}} - I^{2} - \gamma }}{{({\mathbf{Wz}})^{{\text{T}}} {\mathbf{o}}}},$$

in which *γ* will be proved to be the variance of residuals later.

### Intercept and slope

It can be further proved that the value of the regression coefficient representing the slope remains unchanged regardless of whether the intercept is retained in the model. Concretely speaking, if the constant term in Eq. (5) is removed or set as 0, we have the form of Eq. (4). Equations (4) and (5) share the same slope. First of all, let’s examine the model with constant term, Eq. (6). Letting *x* = *z* and *y* = *nWz*, we can apply the formulae of coefficients of univariate linear regressive models to Eq. (6) (Appendix 2). Considering that the mean of *z*_*i*_ is zero, we have,

15 $$b = \frac{{\sum\limits_{i = 1}^{n} {z_{i} (n{\mathbf{Wz}})_{i} } - \left\langle {n{\mathbf{Wz}}} \right\rangle \sum\limits_{i = 1}^{n} {z_{i} } }}{{\sum\limits_{i = 1}^{n} {z_{i}^{2} } }} = \frac{{n\sum\limits_{i = 1}^{n} {z_{i} ({\mathbf{Wz}})_{i} } }}{{\sum\limits_{i = 1}^{n} {z_{i}^{2} } }} = {\mathbf{z}}^{{\mathbf{T}}} {\mathbf{Wz = }}I,$$

where ‹·› represents averaging “·”, *i* = 1,2,3,…,*n*. In Eq. (15), ∑*z*_*i*_ = **z**^T^**o** = 0, ∑*z*_*i*_^2^ = **z**^T^**z** = *n*. This proves that the slope of the spatial autocorrelation model is just Moran’s index from a new perspective. The constant term can be calculated by the means of *z* and *nWz*. Thus we have,

16 $$a = \frac{1}{n}\sum\limits_{i = 1}^{n} {(n{\mathbf{Wz}})_{i} } - b\frac{1}{n}\sum\limits_{i = 1}^{n} {z_{i} } = \sum\limits_{i = 1}^{n} {({\mathbf{Wz}})_{i} } = ({\mathbf{Wz}})^{{\text{T}}} {\mathbf{o}},$$

which indicates that the intercept is just the mean of *nWz*. It can be proved that if Moran’s index *I* = *b* = 1, we have *a* = (**Wz**)^T^o = 0. This suggests the intercept *a* can also reflect spatial autocorrelation. The stronger the autocorrelation, the closer the intercept approaches 0.

Next, let’s examine the model without constant term. This model can be given by revising Eq. (4). The expression is as follows:

17 $${\mathbf{z}}^{{\text{T}}} {\mathbf{zWz}} = n{\mathbf{Wz}} = b{\mathbf{z}} + {\mathbf{e}}^{*} ,$$

where e^*^ represents the residuals term in the zero-intercept model. In this instance, the slope can be given by the ratio of the inner product between **z** and *n***Wz** to the inner product of **z**, that is,

18 $$b = \frac{{\sum\limits_{i = 1}^{n} {z_{i} (n{\mathbf{Wz}})_{i} } }}{{\sum\limits_{i = 1}^{n} {z_{i}^{2} } }} = \frac{{n{\mathbf{z}}^{{\mathbf{T}}} {\mathbf{Wz}}}}{{{\mathbf{z}}^{{\text{T}}} {\mathbf{z}}}}{\mathbf{ = z}}^{{\mathbf{T}}} {\mathbf{Wz = }}I.$$

Comparing Eq. (18) with Eq. (15) shows that the slope of the linear model without intercept is the same as that of the linear model with intercept. This suggests that the existence of intercept does not influence the value of slope, and thus the intercept and slope can be used to describe spatial autocorrelation independently. Especially, the regressive coefficient can be connected to the Eigenvalue of *n***W**.

For the model with constant term, we have two different expressions of the intercept. One is Eq. (14), and the other is Eq. (16). Combining Eqs. (14) and (16) yields,

19 $$a = \frac{{n({\mathbf{Wz}})^{{\text{T}}} {\mathbf{Wz}} - I^{2} - \gamma }}{{({\mathbf{Wz}})^{{\text{T}}} {\mathbf{o}}}} = ({\mathbf{Wz}})^{{\text{T}}} {\mathbf{o}}.$$

From Eq. (19) it follows:

20 $$\gamma = n({\mathbf{Wz}})^{{\text{T}}} {\mathbf{Wz}} - (({\mathbf{Wz}})^{{\text{T}}} {\mathbf{o}})^{2} - I^{2} .$$

On the other hand, the inner product of the residuals vector is,

21 $${\mathbf{e}}^{{\text{T}}} {\mathbf{e}} = (n{\mathbf{Wz}} - a{\mathbf{o}} - b{\mathbf{z}})^{{\text{T}}} (n{\mathbf{Wz}} - a{\mathbf{o}} - b{\mathbf{z}}).$$

Expanding and rearranging Eq. (21) yields (Appendix 3)

22 $${\mathbf{e}}^{{\text{T}}} {\mathbf{e}} = n(n({\mathbf{Wz}})^{{\text{T}}} {\mathbf{Wz}} - (({\mathbf{Wz}})^{{\text{T}}} {\mathbf{o}})^{2} - I^{2} ).$$

The variance of the residuals is obtained by dividing the inner product of *e* by *n*. Comparing Eq. (22) with Eq. (20), and considering Eq. (10), we have

23 $$\gamma = \frac{1}{n}{\mathbf{e}}^{{\text{T}}} {\mathbf{e}} = n({\mathbf{Wz}})^{{\text{T}}} {\mathbf{Wz}} - (({\mathbf{Wz}})^{{\text{T}}} {\mathbf{o}})^{2} - I^{2} = ({\mathbf{Wz}})^{{\text{T}}} {\mathbf{e}} = \sigma_{e}^{2} .$$

where *σ*_*e*_ denotes the population standard deviation of the residuals *e*. Substituting Eq. (23) into Eq. (20) yields

24 $$n({\mathbf{Wz}})^{{\text{T}}} {\mathbf{Wz}} - I^{2} - \sigma_{{\text{e}}}^{2} = (({\mathbf{Wz}})^{{\text{T}}} {\mathbf{o}})^{2} = a^{2} ,$$

which is a useful relation for understanding the boundary values of Moran’s index. Especially, Eq. (24) reflects the relationship between the model’s constant term, *a*, and Moran’s index, *I*.

### Least squares algorithm

The premise of application of a mathematical model to solving real problems lies in effective algorithm. The spatial autocorrelation equation is actually a linear regression model. The principal algorithm of ordinary linear regressive analysis is the least squares method. The above reasoning has involved the least squares method. It can be demonstrated that the least squares principle can be applied to estimation of the values of Moran’s index and related parameters. Based on matrix transformation, the regressive coefficients of Eq. (6) can be computed by

25 $${\mathbf{B}} = \left[ {\left[ {\begin{array}{*{20}c} {{\mathbf{o}}^{{\text{T}}} } \\ {{\mathbf{z}}^{{\text{T}}} } \\ \end{array} } \right]\left[ {\begin{array}{*{20}c} {\mathbf{o}} & {\mathbf{z}} \\ \end{array} } \right]} \right]^{ - 1} \left[ {\begin{array}{*{20}c} {{\mathbf{o}}^{{\text{T}}} } \\ {{\mathbf{z}}^{{\text{T}}} } \\ \end{array} } \right]n{\mathbf{Wz}} = \left[ {\begin{array}{*{20}c} {{\mathbf{o}}^{{\text{T}}} {\mathbf{o}}} & {{\mathbf{o}}^{{\text{T}}} {\mathbf{z}}} \\ {{\mathbf{z}}^{{\text{T}}} {\mathbf{o}}} & {{\mathbf{z}}^{{\text{T}}} {\mathbf{z}}} \\ \end{array} } \right]^{ - 1} \left[ {\begin{array}{*{20}c} {n{\mathbf{o}}^{{\text{T}}} {\mathbf{Wz}}} \\ {n{\mathbf{z}}^{{\text{T}}} {\mathbf{Wz}}} \\ \end{array} } \right],$$

where **B** refers to the coefficient vector. As indicated above, **o**^T^**z** = 0, **o**^T^**o** = **z**^T^**z** = *n*, **z**^T^**Wz** = *I*. According to the principle of linear regression analysis, Eq. (25) can be changed to

26 $${\mathbf{B}} = \left[ {\begin{array}{*{20}c} n & 0 \\ 0 & n \\ \end{array} } \right]^{ - 1} \left[ {\begin{array}{*{20}c} {n{\mathbf{o}}^{{\text{T}}} {\mathbf{Wz}}} \\ {n{\mathbf{z}}^{{\text{T}}} {\mathbf{Wz}}} \\ \end{array} } \right] = \frac{1}{{n^{2} }}\left[ {\begin{array}{*{20}c} n & 0 \\ 0 & n \\ \end{array} } \right]\left[ {\begin{array}{*{20}c} {n{\mathbf{o}}^{{\text{T}}} {\mathbf{Wz}}} \\ {n{\mathbf{z}}^{{\text{T}}} {\mathbf{Wz}}} \\ \end{array} } \right] = \left[ {\begin{array}{*{20}c} {{\mathbf{o}}^{{\text{T}}} {\mathbf{Wz}}} \\ I \\ \end{array} } \right].$$

Apparently, the vector **B** gives two regression parameters: one is the constant term,** o**^T^**Wz**, and the other is the autocorrelation coefficients, *I*. Equation (26) corresponds to Eqs. (15) and (16).

The above process can also be processed by means of determinant operation. Based on Eqs. (11) and (12), three determinants can be constructed as follows:

$$A = \left| {\begin{array}{*{20}c} {nI} & n \\ {n({\mathbf{Wz}})^{{\text{T}}} {\mathbf{Wz}} - \gamma } & I \\ \end{array} } \right|, B = \left| {\begin{array}{*{20}c} 0 & {nI} \\ {({\mathbf{Wz}})^{{\text{T}}} {\mathbf{o}}} & {n({\mathbf{Wz}})^{{\text{T}}} {\mathbf{Wz}} - \gamma } \\ \end{array} } \right|, C = \left| {\begin{array}{*{20}c} 0 & n \\ {({\mathbf{Wz}})^{{\text{T}}} {\mathbf{o}}} & I \\ \end{array} } \right|.$$

In terms of Cramer’s rule in linear algebra, the regressive coefficients can be calculated by the following formulae:

27 $$a = \frac{A}{C} = \frac{{n({\mathbf{Wz}})^{{\text{T}}} {\mathbf{Wz}} - I^{2} - \sigma_{{\text{e}}}^{2} }}{{({\mathbf{Wz}})^{{\text{T}}} {\mathbf{o}}}},$$

28 $$b = \frac{B}{C} = \frac{{ - nI({\mathbf{Wz}})^{{\text{T}}} {\mathbf{o}}}}{{ - n({\mathbf{Wz}})^{{\text{T}}} {\mathbf{o}}}} = I,$$

which are equivalent to the results from matrix transformation. Equations (27) and (28) corresponds to Eqs. (13) and (14). Due to **z**^T^**e** = 0, determinant operation is a little indirect and superfluous where practical application is concerned. However, in theory, combining the matrix transformation results and determinant operation results, we can find another way to derive Eq. (24), which provides a new way of understanding the bounds of Moran’s index.

### Parameter bounds

By analogy with the bounds of Pearson correlation coefficient and autocorrelation coefficients of time series, the bounds of Moran’s index was regarded as coming between − 1 and 1. However, the view may be not correct under different independence assumptions. More than one finding in literature indicates that Moran’s index varies from the minimum Eigenvalue to the maximum Eigenvalue of the* n* times of globally normalized spatial weight matrix^6–11^. This paper complements the criterion from three different angles of view. First, *the angle of spatial weight matrix* (I type). In light of Eqs. (1) and (2), we have

29 $$I = \frac{{{\mathbf{z}}^{{\text{T}}} (n{\mathbf{W}}){\mathbf{z}}}}{{{\mathbf{z}}^{{\text{T}}} {\mathbf{z}}}} = \frac{{{\mathbf{z}}^{{\text{T}}} ({\mathbf{z}}^{{\text{T}}} {\mathbf{zW}}){\mathbf{z}}}}{{{\mathbf{z}}^{{\text{T}}} {\mathbf{z}}}}.$$

According to the principle of Rayleigh quotient^8^, we get the first value range of Moran’s index as follows:

30 $$\lambda_{\min } \le I \le \lambda_{\max } ,$$

in which *λ* denotes the Eigenvalues of *n***W**,* λ*_max_ and *λ*_min_ are the maximum and minimum Eigenvalues of *n***W**, respectively. The bounds values are only determined by the Eigenvalues, and the Eigenvalues are determined only by spatial weight matrix. More than one method results in the inference that the value of Moran’s index is confined by the minimum and maximum Eigenvalues of spatial weight matrix^6–8^.

Second, *the angle of the inner product of spatial weight matrix* (II type). The inner product of spatial weight matrix is **W**^T^**W**. From the inner product equation, Eq. (5), it follows:

31 $$I^{2} + (({\mathbf{Wz}})^{{\text{T}}} {\mathbf{o}})^{2} = \frac{{{\mathbf{z}}^{{\text{T}}} (n^{2} {\mathbf{W}}^{{\text{T}}} {\mathbf{W}}){\mathbf{z}}}}{{{\mathbf{z}}^{{\text{T}}} {\mathbf{z}}}} = \frac{{{\mathbf{z}}^{{\text{T}}} (n{\mathbf{W}})^{{\text{T}}} (n{\mathbf{W}}){\mathbf{z}}}}{{{\mathbf{z}}^{{\text{T}}} {\mathbf{z}}}}.$$

So we have the second value range of parameters as follows:

32 $$\lambda_{\min }^{*} \le I^{2} + (({\mathbf{Wz}})^{{\text{T}}} {\mathbf{o}})^{2} \le \lambda_{\max }^{*} ,$$

where λ^*^ refers to the Eigenvalues of (*n***W)**^T^(*n***W)**, λ^*^_max_ and λ^*^_min_ are the maximum and minimum Eigenvalues of (*n***W)**^T^(*n***W)**, respectively. This represents a pure theoretical expression. In the mathematical world, the error term can be removed, and thus *σ*_*e*_ = 0. However, in the real world, the errors cannot be ignored. Thus, based on Eq. (24), (32) can be revised as,

33 $$\lambda_{\min }^{*} \le I^{2} + (({\mathbf{Wz}})^{{\text{T}}} {\mathbf{o}})^{2} + \sigma_{e}^{2} \le \lambda_{\max }^{*} ,$$

which represents an empirical expression for the second boundary values. The bounds values are determined by the Eigenvalues and spatially weighted vector, and the Eigenvalues are determined only by squared spatial weight matrix. Equations (32) and (33) suggest that the constant term of the spatial autocorrelation equation influences the boundary values of Moran’s index.

Third, *the angle of the outer product of weighted size vector* (III type). The weighted vector is **Wz**, and the corresponding outer product is **Wz**(**Wz**)^T^** = Wzz**^T^**W**. From the outer product equation, Eq. (3), we can derive the following relation,

34 $$({\mathbf{Wz}})^{{\text{T}}} {\mathbf{zz}}^{{\text{T}}} {\mathbf{Wz}} = I({\mathbf{Wz}})^{{\text{T}}} {\mathbf{z}} = I^{2} ,$$

which apparently can be obtained from Eq. (1) by taking square. Considering Eq. (2), we have,

35 $$I^{2} = \frac{{{\mathbf{z}}^{{\text{T}}} (n{\mathbf{W}}^{{\text{T}}} {\mathbf{zz}}^{{\text{T}}} {\mathbf{W}}){\mathbf{z}}}}{{{\mathbf{z}}^{{\mathbf{T}}} {\mathbf{z}}}} = \frac{{{\mathbf{z}}^{{\text{T}}} {\mathbf{W}}^{{\text{T}}} {\mathbf{z}}^{{\mathbf{T}}} {\mathbf{zzz}}^{{\text{T}}} {\mathbf{Wz}}}}{{{\mathbf{z}}^{{\mathbf{T}}} {\mathbf{z}}}}.$$

Then we have the third set of value range of Moran’s index as follows:

36 $$\lambda_{\min }^{**} \le I^{2} \le \lambda_{\max }^{**} ,$$

where λ^**^ denotes the Eigenvalues of *n***W**^T^**zz**^T^**W**, λ^**^_max_ and λ^**^_min_ represent the maximum and minimum Eigenvalues of *n***W**^T^**zz**^T^**W**, respectively. It can be proved that λ^**^_min_ = 0, λ^**^_max_ = *n***(Wz)**^T^**Wz**, which suggests that *I*^2^ ≤ *n***(Wz)**^T^**Wz**. The bounds values are determined by the Eigenvalues, and the Eigenvalues are determined by the outer product of spatially weighted vector.

## Empirical analysis

### Study area and data

This work is devoted to exploring the theoretical foundation of spatial autocorrelation models based on Moran’s index. Nevertheless, the results of mathematical derivation need to be testified by observational data from the real world. If and only if a reasoning result is consistent with the calculated results based on observed data, it is really valid. As we know, the success of natural sciences lies in their great emphasis on the role played by quantifiable data and their interplay with models^12^. So do social sciences to a great degree today. To evaluate the theoretical reasoning results, several sets of observational data will be utilized to verify the models and relations given in Sect. “Theoretical results”. The study area is Beijing-Tianjin-Hebei (BTH) region of China, including Beijing Municipality, Tianjin Municipality, and Hebei Province. It is sometimes termed Jing-Jin-Ji (JJJ) region in literature. The study region includes 13 cities at and above the prefecture level and 22 county-level cities. That is to say, there are 35 cities in total in our study area (*n* = 35). Three sources of observational data are available. The spatial distances are measured by traffic mileage map, and this dataset was extracted by ArcGIS. City sizes were measured by two indicators. One is urban census population in 2000 (the fifth census) and 2010 (the sixth census), and the other is urban nighttime light (NTL) intensity (Table 1). The data of NTL intensity can be reflected by the total number of NTLs within built-up area of cities in the BTH region^13, 14^. The spatial proximity is defined by the inverse function of distance, i.e. *v*_*ij*_ = 1/*r*_*ij*_, where *r*_*ij*_ denotes the traffic mileage between city *i* and city *j*. Therefore, the spatial contiguity matrix can be expressed as **V** = [*v*_*ij*_] = [1/*r*_*ij*_], in which the diagonal elements are defined as zero. That is to say, if *i* = *j*, then *v*_*ij*_ = 0, or else, *v*_*ij*_ = 1/*r*_*ij*_. Globally normalizing **V** results in a spatial weight matrix **W** = **V**/*V*_0_ = [*w*_*ij*_], where *w*_*ij*_ = *v*_*ij*_/*V*_0_, and *V*_0_ = ∑_*i*_∑_*j*_*v*_*ij*_. Obviously, the summation of the entries in **V** does not equal 1, but the summation of the elements in **W** is equal to 1, i.e. the standard spatial weight matrix satisfies the normalization condition, ∑_*i*_∑_*j*_*w*_*ij*_ = 1 (Files S1; S2).

**Table 1:** The measures and data sources for empirical analysis of spatial autocorrelation based on Moran’s index.

Measure	Symbol	Meaning	Data source	Year
Distance	*r*_*ij*_	Interurban distance	Extraction by ArcGIS	2010
City size 1	*x*_*i*_^(1)^	Natural logarithm of city population	The fifth and sixth census of China	2000, 2010
City size 2	*x*_*i*_^(2)^	Natural logarithm of nighttime light (NTL) intensity	American NOAA National Centers for Environmental Information (NCEI)	2000, 2010

### Empirical results and analysis

**Figure 1:**
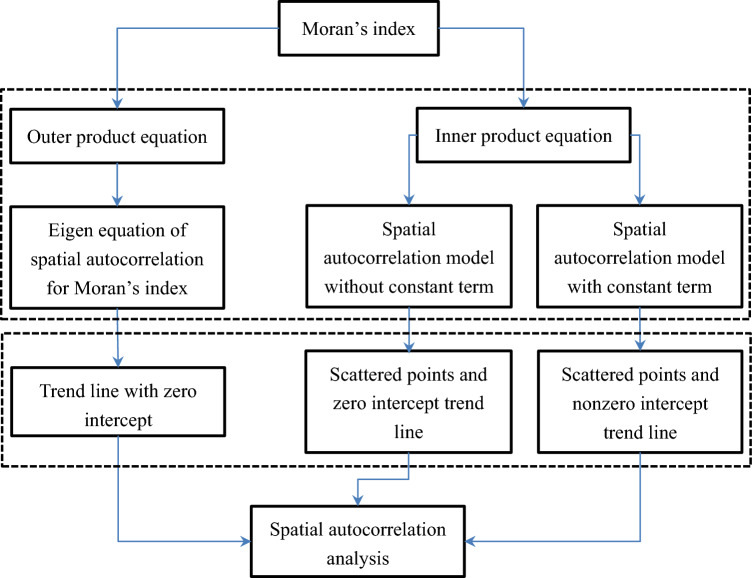
Spatial autocorrelation analysis based on inner product and outer product equations and normalized Moran’s scatterplot. The block diagram show a process of measurement, modeling, scatterplots, and analysis based on the spatial autocorrelation equations.

The following empirical analysis includes three aspects. First, testify the relationships between the parameters of spatial autocorrelation models and Moran’s index. Second, examine the normalized Moran’s scatterplots. Third, verify the key formulae for parameter estimation. The third aspect, i.e. verifying representative relations, is just a simple demonstration for readers. The process of spatial autocorrelation modeling and analysis based on Moran’s index can be illustrated by a flow chart (Fig. 1). The main analytical steps are as follows.

First of all, the parameter values of spatial autocorrelation models are estimated by using the least squares regression analysis. The logarithms of city population and nighttime light intensity are taken as size measures of cities. The reason for taking logarithm is that the hierarchy of city sizes obeys the law of exponential decay. In other words, Zipf’s law and allometric scaling law can be decomposed into two exponential functions^15^. The analysis process consists of five steps as follows. *Step 1*: taking logarithm of a city size variable in given time, say, city population in 2010. Thus, we have *y* = ln(*x*), in which *x* refers to the original variable, and *y* denotes the logarithmic variable. Standardizing *y* yields *z*, which is based on the population standard deviation. *Step 2*: normalizing the spatial contiguity matrix to yield standardized spatial weight matrix. As indicated above, the formula is *w*_*ij*_ = *v*_*ij*_/*V*_0_ = *v*_*ij*_/∑_*i*_∑_*j*_*v*_*ij*_, and *v*_*ij*_ = 1/*r*_*ij*_. *Step 3*: estimating Moran’s index (slope) and the corresponding constant (intercept). Using Eq. (6) to make a linear regressive analysis yields the estimated values of the parameters of spatial autocorrelation models. *Step 4*: generating normalized Moran’s scatterplot. Adding the standard trend line based on Eq. (3) and regressive lines based on Eqs. (4) and (5) to Moran’s scatterplot based on standardized variable and normalized spatial weight matrix can create upgraded Moran’s scatterplot. *Step 5*: verifying typical formulae and useful relations. The main formulae include the constant term and intercept of spatial autocorrelation models. The basic relation is Eq. (24), which is useful in spatial autocorrelation modeling.

Now, let’s invest spatial autocorrelation models. The independent variable is **z**, and the dependent variable is *n***Wz**. So, the intercept is (**Wz**)^T^**o**, and the slope is the Moran’s index, *I*. Taking the constant term into account, for the NTL data in 2010, we have a spatial autocorrelation model as follows

37 $$n(Wz)_{i} = - 0.1427 + 0.1248z_{i} + e_{i} ,$$

where *e*_*i*_ denotes residuals. The goodness of fit is *R*^2^ = 0.2301. Equation (37) corresponds to Eq. (5) and is based on Eq. (6). According to the results of parameter estimation, Moran’s index is about *I* = 0.1248, the constant term is -0.1427, which equals the value of (**Wz**)^T^**o**. This is the mean value of *n***Wz**. Letting the constant term to be zero yields another model as below:

38 $$n(Wz)_{i} = 0.1248z_{i} + e_{i}^{*} ,$$

where *e*_i_^*^ denotes another residuals series. The goodness of fit is *R*^2^ = 0.1769. Equation (38) corresponds to Eq. (4). This model gives the same value of Moran’s index *I* = 0.1248. This verifies the following inference: the existence or no of the constant term in the spatial autocorrelation models does not affect the estimation result of Moran index as a slope. In this way, all the model parameter values and corresponding statistics can be worked out (Table 2).

**Table 2:** The parameter values and related statistics of two types of spatial autocorrelation models based on Moran’s index.

Measure	Year	Spatial autocorrelation model (I), (Eq. (4), without intercept)			*R*^2^	Spatial autocorrelation model (II), (Eq. (5), with intercept)			*R*^2^
		Parameter	Coefficients	*P*-value		Parameter	Coefficients	*P*-value	
City population	2000	–	0	–	0.0345	(**Wz**)^T^**o**	− 0.0839	0.0057	0.0433
		*I*	− 0.0347	0.2778		*I*	− 0.0347	0.2303	
	2010	–	0	–	0.0175	(**Wz**)^T^**o**	− 0.0916	0.0043	0.0223
		*I*	− 0.0260	0.4421		*I*	− 0.0260	0.3914	
Nighttime light	2000	–	0	–	0.1013	(**Wz**)^T^**o**	− 0.1255	0.0020	0.1311
		*I*	0.0837	0.0586		*I*	0.0837	0.0325	
	2010	–	0	–	0.1769	(**Wz**)^T^**o**	− 0.1427	0.0011	0.2301
		*I*	0.1248	0.0106		*I*	0.1248	0.0035	

The values of spatial autocorrelation indexes come between the intervals determined by the maximum and minimum Eigenvalues of spatial weight matrix and the derived matrixes.

**Figure 2:**
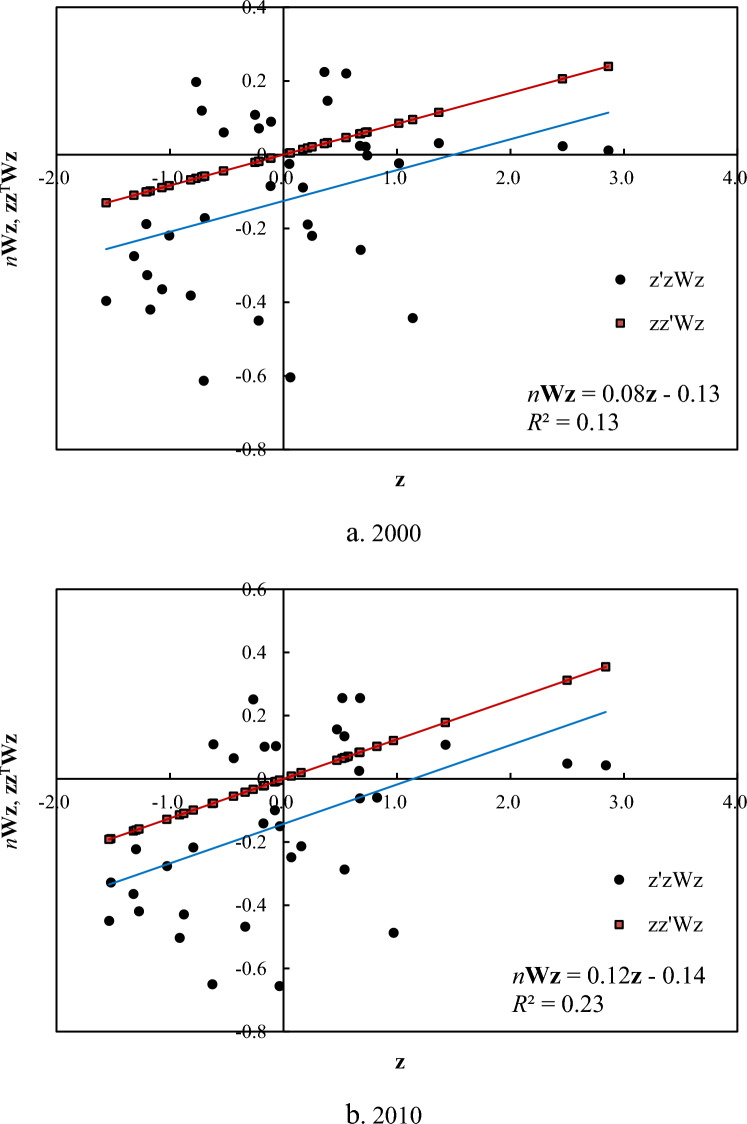
Normalized Moran’s scatterplots for spatial autocorrelation of NTL intensity of cities in Beijing-Tianjin-Hebei region, China. In a normalized Moran’s scatterplot, there are two trend lines. One is based on Eq. (3), and the other is based on Eq. (5). If the trend line is generated by Eq. (4), it will coincide with the trend line generated by Eq. (3). The slope of a trend lines gives the value of Moran’s index, while the intercept of the trend line reflect the mean of spatial weighted standardized variables.

Then, let’s examine the normalized Moran’s scatterplot. Taking the standardized size variable **z** as abscissa axis and *n* times of weighted standardized variable *n***Wz** as ordinate axis, we can generate a Moran’s scatterplot. Still take NTL data as an example. Using Eq. (3) or Eq. (4), we can add the first trend line to the scatterplot; using Eq. (5), we can add the second trend line to the scatterplot. The trend line based on Eq. (3) coincides with the trend line based on Eq. (4), but they bear different statistical meanings. Equation (3) represents an outer product equation and gives a trend line without intercept. Equation (4) represents an inner product equation and gives a trend line with nonzero intercept (Fig. 2). According to Eq. (24), the closer the two trend lines are, the stronger the spatial autocorrelation becomes.

Next, it is necessary to testify the key formulae derived above. Let’s take the NTL intensity data of 2010 as an example. First, three quantities are prepared as follows. The inner product of **Wz** is (**Wz**)^T^(**Wz**) = 0.0025, the mean of *n***Wz** is (**Wz**)^T^**o** = − 0.1427. The variance of model residuals sequence is about 0.0521. In terms of Eqs. (10) and (23), the inner product of the spatially weighted city sizes and autocorrelation model residuals is

39 $$\hat{\gamma } = ({\mathbf{Wz}})^{{\text{T}}} {\mathbf{e}} = \sigma_{e}^{2} = 0.0521.$$

According to Eq. (16), we have the value of constant term in the spatial autocorrelation model as follows:

40 $$\hat{a} = ({\mathbf{Wz}})^{{\text{T}}} {\mathbf{o}} = - 0.1427.$$

According to Eq. (14) or Eq. (19) or Eq. (27), we have the value of model intercept as below:

41 $$\hat{a} = \frac{{n({\mathbf{Wz}})^{{\text{T}}} {\mathbf{Wz}} - I^{2} - \sigma_{{\text{e}}}^{2} }}{{({\mathbf{Wz}})^{{\text{T}}} {\mathbf{o}}}} = \frac{{35 \times 0.0025 - 0.1248^{2} - 0.0521}}{ - 0.1427} = - 0.1427.$$

Clearly, the result based on Eq. (41) is equivalent to the result based on Eq. (40). According to Eq. (13) or Eq. (18) or Eq. (28), we have,

42 $$\hat{b} = {\mathbf{z}}^{{\mathbf{T}}} {\mathbf{Wz = }}0.1248.$$

This is the value of Moran’s index. Finally, we can verify Eq. (24), the results are as below:

43 $$n({\mathbf{Wz}})^{{\text{T}}} {\mathbf{Wz}} - I^{2} - \sigma_{{\text{e}}}^{2} = 35 \times 0.0025 - 0.1248^{2} - 0.0521 = 0.0204,$$

44 $$(({\mathbf{Wz}})^{{\text{T}}} {\mathbf{o}})^{2} = ( - 0.1427)^{2} = 0.0204.$$

This implies that the left side of Eq. (24) is equal to its right side. Other equations or relations can be verified in the similar way by means of observational data.

Although the objective of this study is not the empirical analysis of spatial autocorrelation of cities in BTH region, we might as well look at the principal data analysis conclusions. First, there is nonlinear spatial autocorrelation rather than linear spatial autocorrelation among these cities. For original variables, which are not taken logarithm, the spatial autocorrelation is not significant for the significance level at *α* = 0.05; in contrast, if the size variables are taken logarithm, there will be significant spatial autocorrelation. This suggests that the spatial autocorrelation is based on allometric relationships rather than linear relationships. Second, for different measurements, spatial autocorrelation effects are different. For urban census population, spatial autocorrelation is not significant; in contrast, for urban NTL data, spatial autocorrelation is significant. This indicates that, despite correlation between city population size and urban NTL intensity, the two measures, population and NTL, cannot be replaced with one another. Third, the intercept value does not influence the slope value, i.e. Moran’s *I*. There is a negative correlation relation between the slope values and intercept values. Whether the spatial autocorrelation model retains the constant term or not, the Moran index given by the regression coefficient is not affected. In addition, the model intercept is significant regardless of whether the spatial autocorrelation is significant. Fourth, the spatial autocorrelation evolution trend of urban population sizes and NTL intensity is opposite. From 2000 to 2010, population spatial autocorrelation became weaker, while NTL spatial autocorrelation became stronger. This implies that we can make conventional statistical analysis by means of urban population rather than NTL intensity. After all, the premise of conventional statistical analysis is that there is no significant spatial autocorrelation in a data set. But for NTL datasets, spatial autocorrelation analysis is necessary for BTH region.

Finally, the boundary values of Moran’s index can be analyzed by means of theoretically derived formulae, although the solution to the problem of bounds problem is not the task of this paper. There is a viewpoint in the literature that the boundary values of Moran’s index depends on the maximum and minimum Eigenvalues of the spatial weight matrix, so it is acceptable for the absolute value of the Moran index to exceed 1^6–11^. Let’s examine two real cases. (I) In terms of Eq. (30), the first set of bounds values can be obtained. The bounds values are only determined by spatial weight matrix, **W**^6–9^. For example, based on city population measure in 2000, Moran’s index ranges from − 0.2356 to 1.1118. (II) In terms of Eq. (32) or (33), the second set of bounds values can be worked out. The bounds values are only determined by spatial weight matrix, **W**, and spatial size vector, **z**. But the influence of the size vector is relatively independent. The common influence of spatial weight matrix and size vector on the bounds values is performed through the constant term of spatial autocorrelation equation. For example, based on city population measure in 2000, Moran’s index comes between − 1.1087 and 1.1087. If the residuals variance of spatial autocorrelation model is taken into account, the bounds change into − 1.0966 and 1.0966. (III) In terms of Eq. (36), the third set of bounds values can be figured out. The bounds values are determined by the product of spatial weight matrix, **W**, and spatial size vector, **z**. Product implies coupling relation between **W** and **z**. For example, based on city population measure in 2000, Moran’s index comes between – 0.1866 to 0.1866. The calculation processes are simple and easy to master (Files S1 and S2). Comparing the three types of results of bounds values suggests the characteristics as follows. First, the bounds values of Moran’s index is not only one set. Second, the influence factors of boundary values include spatial weight matrix, **W**, and spatially weighted size vector, **Wz**. Third, the stronger the influence of the spatially weighted size vector, the smaller the range of two boundary values. These results indicate that the real boundary values of Moran’s index are determined by the intersection of the three types of boundary values (Table 3). It can be seen that the boundary values obtained from the Rayleigh quotient are uncertain. A new approach must be taken to find the absolute boundary values of Moran’s index.

**Table 3:** Three types and four sets of boundary values of Moran’s index based on two measures.

Year	Type	Equation	City population		Nighttime light	
			Lower limit	Upper limit	Lower limit	Upper limit
2000	I	Equation (30)	− 0.2356	1.1118	− 0.2356	1.1118
	II	Equation (32)	− 1.1087	1.1087	− 1.1047	1.1047
		Equation (33)	− 1.0966	1.0966	− 1.0835	1.0835
	III	Equation (36)	− 0.1866	0.1866	− 0.2629	0.2629
	Intersection of I, II and III		− 0.1866	0.1866	− 0.2356	0.2629
2010	I	Equation (30)	− 0.2356	1.1118	− 0.2356	1.1118
	II	Equation (32)	− 1.1081	1.1081	− 1.1026	1.1026
		Equation (33)	− 1.0947	1.0947	− 1.0788	1.0788
	III	Equation (36)	− 0.1963	0.1963	− 0.2967	0.2967
	Intersection of I, II and III		− 0.1963	0.1963	− 0.2356	0.2967

The second type of bounds are associated with the constant term or even residuals variance of the spatial autocorrelation model. The intersection represents the minimum range jointly given by four groups of values.

The spatial contiguity matrix in above empirical analysis is based on inverse distance function. There are at least 4 functions that can be used to define spatial weights. If the definition of spatial contiguity matrix is changed, does the above analysis conclusion still hold? The answer is affirmative. The reason is that the quadratic form expression of Moran’s index, Eq. (1), does not change the original definition of Moran’s index (Appendix 4). All the above calculation results, which are displayed in Table 2, are easily verified using the classic calculation formula of Moran index. Moreover, if a step function is employed to replace the inverse distance function to generate a spatial contiguity matrix in binary format, the above models, formulas, and analysis conclusions have not changed in any way (see Files S1 and S2).

## Discussion

As a spatial autocorrelation coefficient, Moran’s index is only a spatial statistic measurement. A measurement is used for description rather than inference. By means of a pair of Eigen equations based on inner product and outer product, we can convert the simple measurement into a set of mathematical models (Table 4). Using the models, we can turn the spatial autocorrelation analysis into linear regression analysis which is more familiar to many scientists. In this way, the spatial analysis process become simpler, but more spatial information can be revealed. As shown above, the inner product equations of Moran’s index can be regarded as spatial autocorrelation equation, which is actually inverse functions of spatial autoregressive models. According to the principle of linear algebra, we can derive the expressions of regressive coefficients. The main findings are as follows. First, the intercept, i.e. constant term, is the sum of the elements in the weighted size vector, that is (**Wz**)^T^**o = ∑**(*n***Wz**)**/***n*** = ∑Wz**, and the slope is just equal to Moran’s index, that is, *b* = *I* = **z**^T^**Wz**. Second, removing the intercept does not change the value of the slope. This confirms the effectiveness of Eq. (4). Third, the square of the model intercept is negatively correlated with the square of the slope. This means that the model intercept can reflect the spatial autocorrelation intensity from different angle of view. Fourth, the inner product equation can be employed to generate two trend lines in a normalized Moran’s scatterplot. The trend line given by the inner product equation without constant term coincides with the trend line given by the Eigen equation based on the outer product. Moreover, combining inner product and outer product equations, we can derive three sets of boundary values for Moran’s index. This is helpful to understand the bounds of Moran’s index from a new perspective.

**Table 4:** Similarities and differences between mathematical models and measurements.

Item	Spatial autocorrelation equation	Moran’s index
Attribute	Model	Measurement
Objective	Imitation of system structure	Characterization of systems
Mathematical form	Function, equation	Formula
Characteristics	There are undetermined parameters, which depend on the algorithm	No undetermined parameters, and the index can be calculated directly
Source	Data analysis or theoretical deduction	Definition or construction
Main function	Description and inference	Description

In the spatial autocorrelation models, both the slope (regression coefficient) and intercept (constant term) can provides spatial autocorrelation information. From Eq. (24), we can derive a relation between the squared intercept and squared slope, that is,

45 $$a^{2} = (({\mathbf{Wz}})^{{\text{T}}} {\mathbf{o}})^{2} = n({\mathbf{Wz}})^{{\text{T}}} {\mathbf{Wz}} - I^{2} - \sigma_{{\text{e}}}^{2} .$$

In the mathematical world, a model has no error. So, the pure theoretical form of Eq. (45) is,

46 $$a^{2} + I^{2} = (({\mathbf{Wz}})^{{\text{T}}} {\mathbf{o}})^{2} + ({\mathbf{z}}^{{\text{T}}} {\mathbf{Wz}})^{2} = n({\mathbf{Wz}})^{{\text{T}}} {\mathbf{Wz}}.$$

This means that there is a negative correlation between the square of the constant term (*a*^2^) and the square of the Moran’s index (*I*^2^). The relationship between slope and intercept involves statistical testing, which will discussed later (Table 5). In particular, the intercept is related to the improvement of the Moran scatter plot. In the improved Moran scatter plot, the information of the constant term can be intuitively reflected by the distance between the two trend lines. The closer the two trend lines are, the stronger the spatial autocorrelation; on the contrary, the more two trend lines deviate from each other, the weaker the spatial autocorrelation. However, the information on intercept is not limited to spatial autocorrelation, but also includes information of spatially weighted variables.

**Table 5:** The slope and intercept of the spatial autocorrelation equation and statistic testing.

Hypothesis	Statement	Equation	Meaning	Test
Null hypothesis	No significant spatial autocorrelation in a system	$n{\mathbf{Wz}} = ({\mathbf{o}}^{{\text{T}}} {\mathbf{Wz}}){\mathbf{o}}$, $a^{2} = n({\mathbf{Wz}})^{{\text{T}}} {\mathbf{Wz}}$	The value of Moran’s *I* can be ignored	Probability value (*P*) is less than given significance level (*α*), e.g. *P* ≤ *α* = 0.05
Alternative hypothesis	There exists significant spatial autocorrelation in as system	$n{\mathbf{Wz}} = ({\mathbf{o}}^{{\text{T}}} {\mathbf{Wz}}){\mathbf{o}} + I{\mathbf{z}}$, $a^{2} + I^{2} = n({\mathbf{Wz}})^{{\text{T}}} {\mathbf{Wz}}$	The value of Moran’s *I* must be considered	Degree of confidence is greater than given threshold value, e.g. (1–*P*) × 100% > 95%

If the probability of the null hypothesis is *P*, the probability of the alternative hypothesis is 1–*P*. Significance level, *α*, represents the threshold value of the probability, *P*. The confidence level of the alternative hypothesis is given by the significance of the null hypothesis, that is, (1–*α*) × 100% > 95%.

The geometric sense of the inner and outer product equations should be explained. Where the outer product equation is concerned, Moran’s index can be the only nonzero Eigenvalue of **zz**^**T**^**W** (Appendix 5). Moran’s index is a spatial statistical measure. Under given conditions, the value of the measure is uniquely determinate. The formulae, Eq. (1), give a certain index value. Equation (3) comes from Eq. (1), and from Eq. (3) we can derive Eq. (1). If Eq. (3) gave more than one nonzero Eigenvalue, then Moran’s index value would be not unique. This contradicts the nature of Moran index defined in Eq. (1). Equation (1) gives one nonzero Eigenvalue, representing the real extension length of the size vector in one direction. In contrast, where the inner product equation is concerned, we cannot prove the uniqueness of the Eigenvalue of *n***W** based on Eq. (4). In fact, Eq. (4) gives *n* nonzero Eigenvalues, representing the maximum extension lengths of the size vector in *n* directions.

Spatial autocorrelation analysis is one of important tools for quantitative analysis of geographical systems. The measures, methods, and theory of spatial autocorrelation has been developed for a long time^6–11,16–26^. Compared with previous studies on the inner product equation of Moran’s index, the novelty of this work is as follows. First, the constant term is taken into account. In previous research, the intercept is ignored^2^. It was proved that intercept is independent of slope. This suggests that whether or not the model has a constant term does not affect the estimation of Moran's index. However, the square of the slope is negatively correlated with the square of the intercept. This means that intercept can assist in spatial autocorrelation analysis. Second, clear calculation formulae of models’ parameters were derived. The mathematical structure of both the intercept and slope was brought to light. Third, a new way of understanding the bounds of Moran’s index were proposed. Based on spatial weight matrix, the inner product of spatial weight matrix, and the outer product of weighted size vector, we have at least three sets of boundary values of Moran’s index. Fourth, normalized Moran’s scatterplots were improved. Two trend lines can be added to a Moran’s scatterplot based on the inner product equation with intercept and the equation without intercept.

The use of regression equations to estimate the Moran index is not an innovation of this work. The basic aim of this study is at understanding spatial autocorrelation from the framework of general mathematical methods associated with quadratic form, Eigen equations, and inner product and outer product expressions. Based on quadratic form, two Eigen equations of spatial autocorrelation, inner and outer product equations, can be constructed. Interpreting the inner product equation based on Moran’s index leads to this study. The novelty of the regression equation presented above lies in methodology rather than theory, and lies in the mathematical structure of the parameters rather than linear expressions (Table 6). The idea of regression analysis based on spatial autocorrelation was presented by quantitative geographers such as Anselin^1^. Suppose that *y* refers to a centralization variable of *x*, and W^*^ to the spatial weight matrix standardized by row. Using the centralization vector **y** as an independent variable, and using the corresponding spatially weighted vector W^*^y as a dependent variable, we can obtain a regression model. The model can give a regression coefficient b^*^ = I^*^ = **y**^T^W^*^y/(**y**^T^**y**), where I^*^ is considered as an approximate estimate of the Moran’s *I*. This expression is based on Rayleigh quotient^8^. However, the calculated results are not consistent with the value of Moran’s index given by the classical algebra formula. The algebra formula can be found in literature^3,5,9,17,21^. In fact, if a spatial dataset is not big, there may be significant errors between the calculation results of Moran’s index and the I^*^ values. In contrast, in this paper, the expression of Moran’s index is based on quadratic form rather than Rayleigh quotient^2^. In Eq. (1), the spatial variable is standardized by *z*-score, and spatial weight matrix is globally normalized. This technique does not change the classical definition of Moran’s index. Therefore, the results of using the new spatial autocorrelation equation to calculate Moran’s index are identical to those given by the classical formula.

**Table 6:** A comparison between two types of regression equations for spatial autocorrelation.

Item		Previous methods	Methods in this paper
Spatial variable		Centralization:$y = x - \mu$	z-score:$z = \frac{x - \mu }{\sigma }$
Spatial weight matrix		Row-standardization: W^*^	Global normalization: **W** = **V**/*V*_0_
Moran’s *I*		Rayleigh quotient: $I^{*} = \frac{{{\mathbf{y}}^{{\text{T}}} {\mathbf{W}}^{*} {\mathbf{y}}}}{{{\mathbf{y}}^{{\text{T}}} {\mathbf{y}}}}$	Quadratic form: $I = {\mathbf{z}}^{{\text{T}}} {\mathbf{Wz}}$
Regression model	No intercept	${\mathbf{W}}^{*} {\mathbf{y}} = b^{*} {\mathbf{y}} = I^{*} {\mathbf{y}}$	$n{\mathbf{Wz}} = b{\mathbf{z}} = I{\mathbf{z}}$
	With intercept	$$\begin{gathered} {\mathbf{W}}^{*} {\mathbf{y}} = a^{*} + b^{*} {\mathbf{y}} \\ = a^{*} + I^{*} {\mathbf{y}} \\ \end{gathered}$$	$$\begin{gathered} n{\mathbf{Wz}} = a + b{\mathbf{z}} \\ = a + I{\mathbf{z}} \\ \end{gathered}$$
Intercept		$a^{*} = \frac{1}{n}({\mathbf{Wy}})^{{\text{T}}} {\mathbf{o}}$	$a = ({\mathbf{Wz}})^{{\text{T}}} {\mathbf{o}}$
Slope		$b^{*} = I^{*} = \frac{{{\mathbf{y}}^{{\text{T}}} {\mathbf{W}}^{*} {\mathbf{y}}}}{{{\mathbf{y}}^{{\text{T}}} {\mathbf{y}}}}$	$b = I = {\mathbf{z}}^{{\text{T}}} {\mathbf{Wz}}$
Example: Value of Moran’s *I*	CP2000	– 0.0454	− 0.0347
	CP2010	− 0.0361	− 0.0260
	NTL2000	0.0645	0.0837
	NTL2010	0.1080	0.1248

The previous models were formulated by the author according to the ideas from Anselin^1^.

The spatial weight matrix, **W**, is globally normalized, and W^*^ is normalized by row. Correspondingly, **V** denotes spatial contiguity matrix of no normalization, and *V*_0_ is the sum of entries in **V**. *x* denotes the original variable, *μ* is mean, and *σ* is standard deviation.

“CP2000” means city population in 2000, “CP2010” means city population in 2010, “NTL2000” means nighttime light in 2000, and “NTL2010” means nighttime light in 2010.

As a mathematical model, spatial autocorrelation equations require certain algorithms. On the other hand, as a spatial statistical analysis method, model parameters involve statistical testing. The algorithm of this study is based on the classical framework of least squares technology. The corresponding statistical tests also come from this framework. The spatial autocorrelation models conform to all the classical assumptions in linear regression analysis, but its significance based on *t*-test is not necessarily reliable^1^. The *P*-value given in the empirical analysis of this paper is based on conventional linear analysis. But this is just a stopgap measure, after all, this work is a theoretical and methodological study rather than a practical analysis. It can be seen that for the same Moran's index value, considering the model intercept and ignoring the model intercept result in different *P*-values (Table 2). In fact, spatial autocorrelation analysis leads to a paradox: If a spatial dataset does not have significant spatial autocorrelation, then the Moran’s index and the corresponding statistical tests of correlation are reliable; on the contrary, if a spatial dataset has significant spatial autocorrelation, the Moran’s index and statistical tests of correlation are unreliable. The calculation of the Moran’s index relies on mean values, while mean values rely on numerical summation calculations. If there is no spatial autocorrelation, the sum results can reflect the overall information content of the system, so the means are valid; conversely, if there is significant spatial autocorrelation, the whole is not equal to the sum of parts, and the numerical summation results exhibit information affine phenomenon. In this case, the average value is unreliable, resulting in issues with the Moran’s index and corresponding statistical tests.

The Eigen equation provides a new framework for spatial autocorrelation analysis and opens up a new perspective for the development of spatial analysis theory. Based on quadratic form, inner product and outer product, the core of many important mathematical methods can be abstracted as a pair of Eigen equations (Table 7). Through Eigen equations, spatial statistical measures can be transformed into spatial analysis models. By comparing different methods, we can deepen our understanding of spatial autocorrelation analysis. In particular, based on normalized variables and normalized weight matrixes, Getis-Ord’s index can also be expressed as two Eigen equations based on inner product and outer product^27^. This suggests that the mathematical derivation process for spatial statistic parameters can be generalized to Getis-Ord’s index by analogy. Getis-Ord’s index reflects spatial autocorrelation from another geographical angle of view^28^.

**Table 7:** The inner production and outer product equations for a type of mathematical methods.

Type	Method	Inner product	Outer product
Spatial statistics	Moran’s index	${\mathbf{z}}^{{\text{T}}} {\mathbf{zWz}} = n{\mathbf{Wz}} = I{\mathbf{z}}$	${\mathbf{zz}}^{{\text{T}}} {\mathbf{Wz}} = I{\mathbf{z}}$
	Getis-Ord’s index	${\mathbf{p}}^{{\text{T}}} {\mathbf{pWp}} = \xi {\mathbf{Wp}} = G{\mathbf{p}}$	${\mathbf{pp}}^{{\text{T}}} {\mathbf{Wp}} = G{\mathbf{p}}$
Multivariable statistics	Principal component analysis (PCA) and factor analysis (FA)	$(\frac{1}{n}{\mathbf{X}}^{{\text{T}}} {\mathbf{X}}){\mathbf{a}} = {\mathbf{Ra}} = \lambda {\mathbf{a}}$	$(\frac{1}{n}{\mathbf{XX}}^{{\text{T}}} ){\mathbf{f}} = \lambda {\mathbf{f}}$
System analysis	Analytic hierarchy process (AHP)	$({\mathbf{WW}}_{*}^{{\text{T}}} ){\mathbf{W}} = n{\mathbf{W}}$	${\mathbf{W}}({\mathbf{W}}_{*}^{{\text{T}}} {\mathbf{W}}) = n{\mathbf{W}}$

For Getis-Ord’s index *G*, **p** denotes a normalized vector, *ξ* = **p**^T^**p** refers to the inner product of **p**. For PCA and FA, **X** denotes standardized matrix, **R** is Pearson correlation matrix, *λ* is an Eigenvalue, **a** and **f** refer to Eigenvectors. For AHP method, **W** denotes weight vector, W_*_ is the reciprocal vector of **W**.

The main shortcomings of this study lie in three aspects. First, the spatial autoregressive modeling corresponding to the spatial autocorrelation model has not be discussed. Correlation and regression represent two different sides of the same coin in statistics, and spatial autoregressive modeling depends on spatial autocorrelation analysis. In fact, the spatial autocorrelation models can be treated as inverse spatial autoregressive models. In this sense, the spatial autocorrelation equations can serve as a theoretical bridge between spatial autocorrelation measures and spatial autoregressive modeling. Second, no conclusion has been reached on the controversial issue of the boundary value of Moran’s index. This work only provides a new perspective on understanding the boundary values of the Moran index from three expressions of Rayleigh quotient. Third, the problem of significance testing for Moran’s index has not been solved. Because of spatial autocorrelation paradox, the *P*-value based on conventional regression analysis are not convincing. As mentioned earlier, using ordinary regression analysis methods to calculate *P*-values is a stopgap approach. Due to the limitations of space and topic, the three problems will be explored in companion papers.

## Conclusions

The spatial autocorrelation models based on inner product equation and outer product equation of Moran’s index are useful in both theoretical development and empirical research of geographical analysis. From the results of mathematical derivation and positive study, the chief conclusions can be drawn as follows. *First, a spatial autocorrelation model is an inner product equation based on Moran’s index, and the equation is actually an inverse function of the simplest spatial autoregressive model*. Spatial autocorrelation differs from temporal autocorrelation, so spatial autoregressive process differs from temporal autoregressive process. The difference lies in spatial symmetry and temporal asymmetry. A number of models and methods of spatial analysis were actually developed by analogy with time series analysis. However, time series autocorrelation is a unidirectional process defined in 1-dimensional time, while spatial autocorrelation is a bidirectional process defined in 2-dimensional space. Therefore, a time autoregressive model has no inverse function, while a spatial autoregressive model has an inverse function, that is, spatial autocorrelation model. *Second, inner product equations can be employed to improve normalized Moran’s scatterplot*. Based on standardized variable and globally normalized weight matrix, spatial autocorrelation models for Moran’s index have a pair of expressions: one is that bears constant term, and the other is that bears no constant term. The existence of constant term does not affect the autocorrelation coefficient of the model. This proves that the expression of the inner product equation is valid. Using the pair of linear regression models based on the inner product equation, we can improve normalized Moran’s scatterplot by adding two trend lines. The trend line based on the zero-intercept autocorrelation model coincides with the standard trend line based on outer product equation, and the trend line based on the autocorrelation model bearing intercept deviates the standard trend line. The slopes of the trend lines give the value of Moran’s index, and the distance between the two trend lines reflects the intensity of spatial autocorrelation. *Third, the boundary values of Moran’s index depend on the relationships between spatial weight matrix and size vector*. Some scholars believe that the boundary values is − 1 and 1, while others believe that it is determined by the maximum and minimum Eigenvalues of the spatial weight matrix. However, the actual situation is not so simple. Based on the simplified formula of Moran’s index, inner product equation, and outer product equation, we can derive three sets of boundary values. These sets of boundary values are not equivalent to one another. The first one is based on spatial weight matrix, the second one is based on the inner product of the spatial weight matrix, and the third one is based on the outer product of weighted size vector. The actual boundary values must be the intersection of these three sets of boundary values. *Fourth, the regressive analysis based on least squares method can be used to calculate Moran’s index and related parameters*. The inner product equations of Moran’s index can be treated as spatial autocorrelation models. We have at least four ways of deriving the models’ parameters by using the least squares method. The first is solving algebraic equations, the second is regressive coefficient formulae, the third is matrix manipulation, and the fourth is determinant operation based on Cramer’s rule. The expressions of parameters given by different methods may be different in form, but the results are equivalent to one another.

### Supplementary Information

Supplementary Information 1.

Supplementary Information 2.

Supplementary Information 3.

Supplementary Legends.

## Data Availability

All data generated or analysed during this study are included in its supplementary information.
