# Supplementary material for: Spatial autocorrelation equation based on Moran’s index
Source: Sci Rep. 2023 Nov 7;13:19296. doi: 10.1038/s41598-023-45947-x (PMC10630413; doi:10.1038/s41598-023-45947-x)
Supplement: Supplementary file 3 — Supplementary Information 3. [file 41598_2023_45947_MOESM3_ESM.docx]

## Supplementary Material

### Appendix 1. Eigen equation and characteristic length

One of the key points of scientific research methods is to find the invariance under transformations and the commensurability of invariants. Consider an operator for transformation, **T**, and a function, *f*(*x*), where *x* is a random variable. If the transformation **T** is applied to the function *f*(*x*) and the structure of the function remains unchanged, then we have

$\mathbf{T}f(x)=\lambda f(x)$. (A1)

Thus, *f*(*x*) is regarded as an *eigen function* of **T**, and the parameter *λ* is an *eigenvalue*. An eigen function is a solution to an eigen equation. The eigenvalue is also termed *characteristic value*, which reflects the characteristic scale of quantitative analysis based on the eigen function. A characteristic scale is often called *characteristic length* because it is always a 1-dimension measure. A large amount of theoretical modeling and data analysis are based on the invariance principle in a transformation shown in equation (A1). For example, a fractal model is an eigen function under contraction or dilation transformation, while the fractal dimension is associated with the corresponding eigenvalues. In geography, Clark’s model on urban population density model is eigen function of translational and differential transformation, while Smeed’s model on urban traffic network density is eigen function of scaling transformation. According to eigenvalues, we can determine characteristic lengths or characteristic parameters for spatial analysis.

In linear algebra, if a vector is multiplied by a matrix and the result is a constant multiplied by this vector, then the vector is one of the eigenvectors and the constant represents the corresponding eigenvalues. The invariant relationship under a matrix transformation can be expressed as

$\mathbf{Mx}=\lambda\mathbf{x}$, (A2)

where **M** denotes a matrix, say, correlation coefficient matrix, **x** is a vector, and *λ* is a parameter. Equation (A2) represents an eigen equation in linear algebra. The **M** and **x** in equation (A1) correspond to **T** and *f*(*x*) in equation (A2). The meaning of equation (A2) is that by applying a transformation to **x** using matrix **M**, there is no essential change in the structure of **x**, but the geometric sizes changes. So, **x** is the eigenvector of **M**, and *λ* is the corresponding eigenvalue. Solving the characteristic polynomial equation of **M** yields the eigenvalues of **M**, so eigenvalue is also termed *characteristic value* or *characteristic root*. Many mathematical methods such as principal component analysis (PCA), factor analysis (FA), and analytic hierarchy process (AHP) are based on the invariance principle in the linear transformation shown in equation (A2). In this paper, two eigen equations are presented. Based on the outer product of **z**, the eigen equation is

$(\mathbf{zz}^{\text{T}}\mathbf{W})\mathbf{z}=I\mathbf{z}$; (A3)

Based on the inner production of **z**, the eigen equation is

$(\mathbf{z}^{\text{T}}\mathbf{zW})\mathbf{z}=(n\mathbf{W})\mathbf{z}=I\mathbf{z}$. (A4)

In theory, Moran’s index is the maximum eigenvalue of spatial correlation matrix, **zz**^T^**W**. In empirical analysis, Moran’s index is associated with the eigenvalues of spatial weight matrix, **W**.

### Appendix 2. The formulae of intercept and slope of linear regressive models

In theory, a univariate linear regression model with a constant term can be expressed as

$y=a+bx$, (B1)

where *x* denotes independent variable, *y* refers to dependent variable. As for the parameters, *a* is the intercept, and *b* is the slope. From equation (B1), we can derive the following relation

$\bar{y}=a+b\bar{x}$, (B2)

where$\bar{x}$and$\bar{y}$represent he average values of the independent and dependent variables. Suppose that the independent variable is a standardized variable by *z*-score. In this case, the mean of the independent variable is 0. By means of the least squares method, the slope can be calculated by

$b=\frac{\sum_{i=1}^{n} (x_{i}-\bar{x})(y_{i}-\bar{y})}{\sum_{i=1}^{n} (x_{i}-\bar{x})^{2}}=\frac{\sum_{i=1}^{n} x_{i}(y_{i}-\bar{y})}{\sum_{i=1}^{n} x_{i}^{2}}=\frac{\sum_{i=1}^{n} x_{i}y_{i}-\bar{y}\sum_{i=1}^{n} x_{i}}{\sum_{i=1}^{n} x_{i}^{2}}$. (B3)

According to equation (B2), the intercept should be

$a=\bar{y}-b\bar{x}=\bar{y}$. (B4)

This suggests that the intercept is equal to the mean of dependent variable. Assuming *x*=**z** and *y*=*n***Wz**, we can derive the following result: *a*=∑*_i_*(**Wz**)*_i_*, *b*=*I*, where *I* denotes Moran’s index. If the constant term in equation (B1) is zero (*a*=0), the sum of *x_i_* is also equal to zero (∑*_i_x_i_*=0), we have $\bar{y}$=*a*=0, then the formula of calculation formula for the slope will change to

$b=\frac{\sum_{i=1}^{n} x_{i}y_{i}}{\sum_{i=1}^{n} x_{i}^{2}}$. (B5)

Substituting *x*=**z** and *y*=*n***Wz** into equation (B5) yields *b*=*I*.

### Appendix 3. The inner product of residuals of a spatial autocorrelation model

The process of derivation and transformation of the inner product equation of residuals in details is as follows:

$\begin{matrix} \mathbf{e}^{\text{T}}\mathbf{e}=(n\mathbf{Wz}-a\mathbf{o}-b\mathbf{z})^{\text{T}}(n\mathbf{Wz}-a\mathbf{o}-b\mathbf{z}) \\ =(n(\mathbf{Wz})^{\text{T}}-a\mathbf{o}^{\text{T}}-b\mathbf{z}^{\text{T}})(n\mathbf{Wz}-a\mathbf{o}-b\mathbf{z}) \\ =n^{2}(\mathbf{Wz})^{\text{T}}\mathbf{Wz}-2an(\mathbf{Wz})^{\text{T}}\mathbf{o}-2nb(\mathbf{Wz})^{\text{T}}\mathbf{z}+a^{2}n+b^{2}\mathbf{z}^{\text{T}}\mathbf{z} \\ =n^{2}(\mathbf{Wz})^{\text{T}}\mathbf{Wz}-2an(\mathbf{Wz})^{\text{T}}\mathbf{o}-2nbI+a^{2}n+b^{2}n \\ =n^{2}(\mathbf{Wz})^{\text{T}}\mathbf{Wz}-2n((\mathbf{Wz})^{\text{T}}\mathbf{o})^{2}-nI^{2}+a^{2}n \\ =n^{2}(\mathbf{Wz})^{\text{T}}\mathbf{Wz}-2n((\mathbf{Wz})^{\text{T}}\mathbf{o})^{2}-nI^{2}+n((\mathbf{Wz})^{\text{T}}\mathbf{o})^{2} \\ =n^{2}(\mathbf{Wz})^{\text{T}}\mathbf{Wz}-n((\mathbf{Wz})^{\text{T}}\mathbf{o})^{2}-nI^{2} \\ =n(n(\mathbf{Wz})^{\text{T}}\mathbf{Wz}-((\mathbf{Wz})^{\text{T}}\mathbf{o})^{2}-I^{2}) \end{matrix}$. (C1)

Therefore, we have

$\frac{1}{n}\mathbf{e}^{\text{T}}\mathbf{e}=n(\mathbf{Wz})^{\text{T}}\mathbf{Wz}-((\mathbf{Wz})^{\text{T}}\mathbf{o})^{2}-I^{2}$, (C2)

which is just the variance of residuals series.

### Appendix 4. Moran’s *I*: from Rayleigh quotient to quadratic form

It can be proved that the quadratic form expression of Moran’s index does not change its original definition. In literature, Moran’s index is often expressed as the form of Rayleigh quotient based on centralization variable. Suppose the original spatial size variable is *x*. The centralization variable is

$y=x-\mu$, (D1)

where *μ* denotes means of *x*. Accordingly, the standardized variable based on *z*-score is

$z=\frac{y}{\sigma}=\frac{x-\mu}{\sigma}$, (D2)

where *σ* denotes population standard deviation of *x*. The matrix form of variance of *x* is

$\sigma^{2}=\frac{1}{n}\mathbf{y}^{\text{T}}\mathbf{y}=\frac{1}{n}(\mathbf{x}-\mu)^{\text{T}}(\mathbf{x}-\mu)$. (D3)

Based on the centralization variable, Moran’s *I* can be expressed as the form of Rayleigh quotient:

$I=\frac{n}{V_{0}}\frac{\mathbf{y}^{\text{T}}\mathbf{Vy}}{\mathbf{y}^{\text{T}}\mathbf{y}}$, (D4)

where *n* denotes the number of spatial elements, **V** refers to spatial contiguity matrix, and *V*_0_ is the sum of entries of **V**. The spatial contiguity matrix, **V**, is always treated as non-normalized spatial weight matrix. So, globally normalized spatial weight matrix is **W**=**V**/*V*_0_. Thus we have

$I=\frac{\mathbf{y}^{\text{T}}(\mathbf{V}/V_{0})\mathbf{y}}{\frac{1}{n}\mathbf{y}^{\text{T}}\mathbf{y}}=\frac{\mathbf{y}^{\text{T}}\mathbf{Wy}}{\sigma^{2}}=(\frac{\mathbf{y}}{\sigma})^{\text{T}}\mathbf{W}(\frac{\mathbf{y}}{\sigma})=\mathbf{z}^{\text{T}}\mathbf{Wz}$, (5)

which is a quadratic form of Moran’s index, i.e., equation (1) in the text.

### Appendix 5. Moran’s *I* is the only one nonzero eigenvalue of matrix zz^T^W

For general cases, a spatial weight matrix is a symmetric matrix because it is based on generalized distance matrix. That is, **W**^T^=**W**. Otherwise, it will violate the distance axiom. Let

$\alpha=\mathbf{z}=\left[\begin{matrix} \alpha_{1} & \alpha_{2} & \cdots& \alpha_{n} \end{matrix}\right]^{\text{T}}$, $\beta=\mathbf{Wz}=\left[\begin{matrix} \beta_{1} & \beta_{2} & \cdots& \beta_{n} \end{matrix}\right]^{\text{T}}$, (E1)

where *α* and *β* denote two vectors, and *α_i_* and *β_i_* refer to the entries of these vectors (*i*=1,2,…,*n*). The expression of outer product of *α* and *β* is as below:

$\mathbf{M}=\alpha\beta^{\text{T}}=\mathbf{zz}^{\text{T}}\mathbf{W}=\left[\begin{matrix} \alpha_{1} \\ \alpha_{2} \\ \vdots\\ \alpha_{n} \end{matrix}\right]\left[\begin{matrix} \beta_{1} & \beta_{2} & \cdots& \beta_{n} \end{matrix}\right]=\left[\begin{matrix} \alpha_{1}\beta_{1} & \alpha_{1}\beta_{2} & \cdots& \alpha_{1}\beta_{n} \\ \alpha_{2}\beta_{1} & \alpha_{2}\beta_{2} & \cdots& \alpha_{2}\beta_{n} \\ \vdots& \vdots& \ddots& \vdots\\ \alpha_{n}\beta_{1} & \alpha_{n}\beta_{2} & \cdots& \alpha_{n}\beta_{n} \end{matrix}\right]$. (E2)

Accordingly, the inner product expression is

$(\beta^{\text{T}}\alpha)=(\mathbf{Wz})^{\text{T}}\mathbf{z}=\mathbf{z}^{\text{T}}\mathbf{Wz}=I$. (E3)

Now, we can calculate

$\mathbf{M}^{2}=(\alpha\beta^{\text{T}})(\alpha\beta^{\text{T}})=\alpha(\beta^{\text{T}}\alpha)\beta^{\text{T}}=(\beta^{\text{T}}\alpha)\alpha\beta^{\text{T}}=I\mathbf{M}$. (E3)

Thus we have

$\mathbf{M}^{2}-I\mathbf{M}=\mathbf{M}(\mathbf{M}-I\mathbf{E})=\mathbf{O}$. (E4)

where **E** is an identity matrix, and **O** is zero matrix. Equation (E4) suggests that the minimum polynomial of matrix **M** is

$f(x)=x^{2}-Ix=x(x-I)=0$, (E4)

which gives unique nonzero root, *x*=*I*. According to algebra theory, the minimum polynomial of a matrix has exactly the same roots as the characteristic polynomial, the matrix **M** has only one characteristic root, i.e., Moran’s *I*. The other characteristic roots are all zero. The characteristic roots of a characteristic polynomial are just the eigenvalues of corresponding matrix. [q.e.d]
