# Supplementary material for: Spatial autocorrelation equation based on Moran’s index
Source: Sci Rep. 2023 Nov 7;13:19296. doi: 10.1038/s41598-023-45947-x (PMC10630413; doi:10.1038/s41598-023-45947-x)
Supplement: Supplementary file 4 — Supplementary Legends. [file 41598_2023_45947_MOESM4_ESM.docx]

## Supplementary information files

**File S1. Spatial autocorrelation modeling processes and results for BTH cities in 2000.** This file contains the original or preliminarily processed data of urban system in the study area of 2000 used in this paper. It provides four complete processes of computing spatial autocorrelation coefficients based on inverse distance function and step function. Three groups of boundary values are displayed, and normalized Moran’s scatterplots are illustrated. (XLSX)

**File S2. Spatial autocorrelation modeling processes and results for BTH cities in 2010**. This file contains the original or preliminarily processed data of urban system in the study area of 2010 used in this paper. (XLSX)
